# Operative treatment of fragility fractures of the pelvis is connected with lower mortality. A single institution experience

**DOI:** 10.1371/journal.pone.0253408

**Published:** 2021-07-09

**Authors:** Pol Maria Rommens, Mehdi Boudissa, Sven Krämer, Miha Kisilak, Alexander Hofmann, Daniel Wagner

**Affiliations:** 1 Department of Orthopaedics and Traumatology, University Medical Center, Mainz, Germany; 2 Department of Orthopaedics and Traumatology, Westpfalz Clinics Kaiserslautern, Kaiserslautern, Germany; University Hospital Zurich, SWITZERLAND

## Abstract

**Background:**

Fragility fractures of the pelvis (FFP) represent an increasing clinical entity. Until today, there are no guidelines for treatment of FFP. In our center, recommendation for operative treatment was given to all patients, who suffered an FFP type III and IV and to patients with an FFP type IIwith unsuccessful non-operative treatment. We performed a retrospective observational study and investigated differences between fracture classes and management alternatives. We hypothetized that operative treatment may reduce mortality.

**Materials and methods:**

The medical charts and radiographs of 362 patients were analysed. Patient demographics, FFP-classification, length of hospital stay (LoS), type of treatment, general and surgery-related complications, mortality, Short Form-8 physical component score (SF-8 PCS) and mental component score (SF-8 MCS), Parker Mobility Score (PMS) and Numeric Rating Scale (NRS) were documented.

**Results:**

238 patients had FFP type II and 124 FFP type III and IV. 52 patients with FFP type II (21.8%) and 86 patients with FFP type III and IV (69.4%) were treated operatively (p<0.001). Overall mortality did not differ between the fracture classes (p = 0.127) but was significantly lower in the operative group (p<0.001). Median LoS was significantly higher in FFP type III and IV (p<0.001) and in operated patients (p<0.001). There were more in-hospital complications in patients with FFP type III and IV (p = 0.001) and in the operative group (p = 0.006). More patients of the non-operative group were mobile (p<0.001) and independent (p<0.001) at discharge. Half of the patients could not return in their living environment.203 of the 235 surviving patients (86%) answered the questionnaires after a mean follow-up time of 38 months. SF-8 PCS, SF-8 MCS and PMS did not differ between the fracture classes and treatment groups. Pain perception was higher in the operated group (p = 0.013).

**Conclusion:**

In our study, we observed that operative treatment of FFP provides low mortality rates, although LoS and in-hospital complications were higher in the operative group. At discharge, the non-operative group was more mobile and independent. At follow up, quality of life and mobility were comparable between the groups. Further prospective studies are needed to clarify the impact of operative treatment of FFP on mortality and functional outcome.

## Introduction

**Fig 1:**
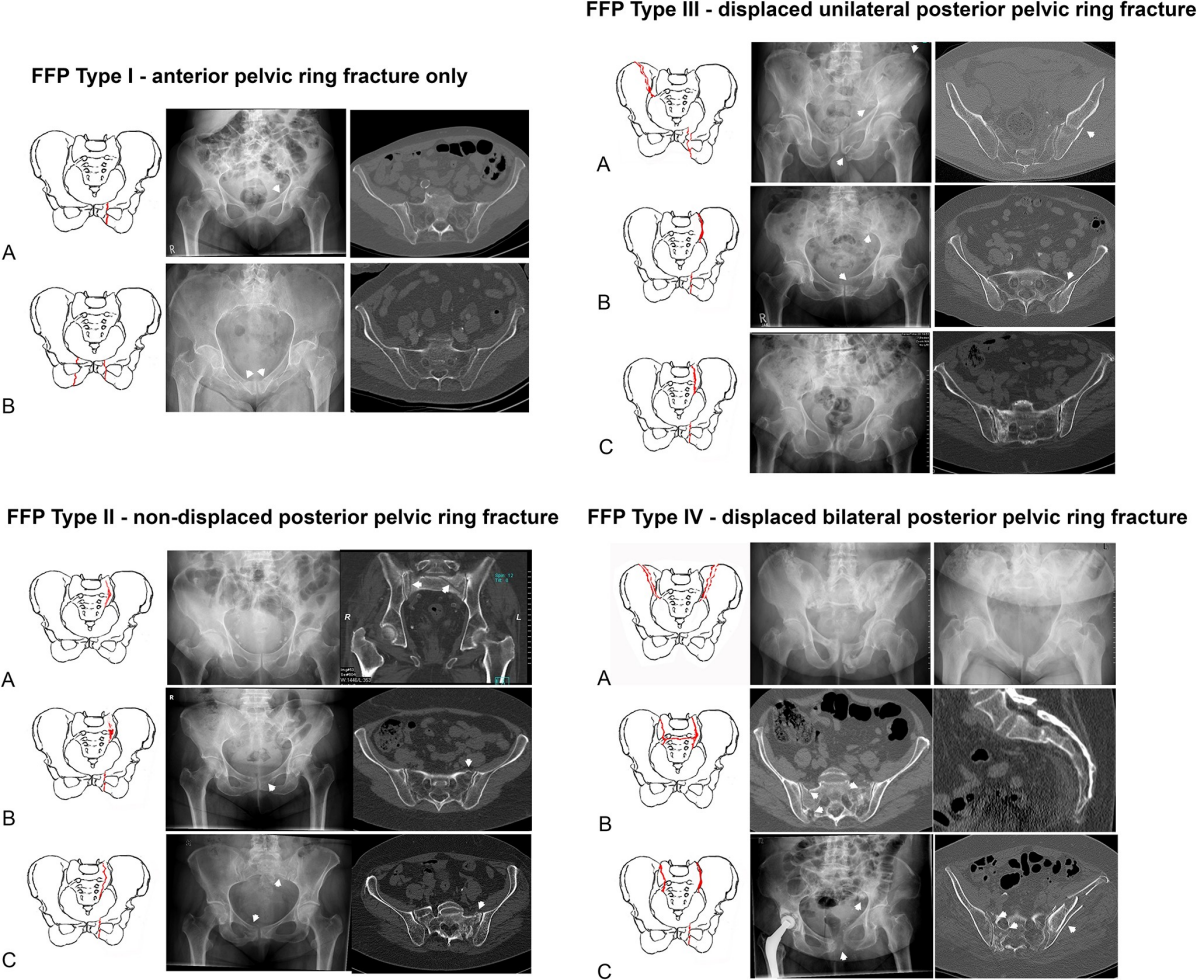
FFP-classification with categories and subcategories [7].

Fragility fractures of the pelvis (FFP) are clinical entities with an increasing incidence among elderly persons [1–3]. Thanks to better prevention and optimized medical care, incidence of elderly persons has been rising in first world countries. The percentage of persons older than 65 years has grown from 16 to 21% between 1994 and 2019 in Germany [4]. Diseases are frequent companions of old age, among them is osteoporosis one of the most common [5]. Due to loss of bone mineral density, the bone structure becomes weaker and proner to fractures [6]. Compared to typical osteoporotic fractures, FFP are less common but very debilitating. Because of different characteristics and outcome between high-energy and low-energy pelvic fractures, a specific FFP-classification, which distinguishes between four categories of increasing instability, was developed. FFP type I includes anterior pelvic ring fractures only, FFP type II non displaced posterior fractures, FFP type III displaced unilateral posterior fractures and FFP type IV displaced bilateral posterior fractures. The subcategories represent different localisations of fractures within each category (Fig 1) [7, 8].

There is an ongoing debate on the most appropriate treatment of FFP. Management should focus on adequate pain relief and early mobilisation. If an operative treatment is chosen, the procedure should be as minimal invasive as possible [9]. FFP type I do not need surgical intervention [10]. Despite the recommendations for surgical treatment, which have been published by the authors in 2013 [7], it is not evident to date that patients with FFP type II, III and IV profit from operative therapy. Recent data from Japan suggest that non-operative treatment is feasable with good functional outcome. Other authors published promising results after operative treatment [11–14]. Most publications focus on small patient cohorts or one specific operative technique. The primary objective of this retrospective observational study was the comparison of mortality between non-operative and operative management of FFP with involvement of the posterior pelvis (FFP type II, III and IV) in a larger patient population. The secondary objective was the analysis of in-hospital complications and patient-related outcome.

## Patients and methods

We retrospectively reviewed medical charts of all adult patients with a pelvic fracture at our level I trauma center between 2005 and mid-2018 (13.5-year period). Excluded were patients with pelvic fractures due to high-energy trauma, with acetabular fractures, with pathologic fractures due to malignancy and with an outpatient treatment. Conventional radiographs and CT were analysed and the fractures classified in accordance to the FFP-classification [7]. Consecutively, patients with FFP type I were excluded, leaving only patients with FFP of the types II, III and IV. We distinguished between patients undergoing non-operative and operative treatment. The patients were asked to agree with the recommendations for surgical treatment described in the publication of 2013 [7]. Surgery was performed after informed and written consent of all patients or their relatives.

The following demographic data were collected at admission: age, sex, living environment and comorbidities. A comorbidityies was only registered as such when the disease was mentioned in the medical history of the discharge letter of the patient. The medical charts were analysed for the following information: FFP-classification of injury, type of management (non-operative versus operative), length of hospital stay (LoS), general in-hospital complications, surgery-related complications and mortality. Mobility at discharge and destination at discharge were documented. Patients, who were able to walk in the room or on the floor were defined as “walkers”, patients who were bedridden or only able to perform transfers from bed to chair as “non walkers”. Patients, who returned home after discharge were regarded as “independent”, the others as “dependent”.

Patients or their relatives were contacted by phone asking for participation in the survey. Their general practitioner or the bureau of vital statistics was contacted to ask about their vital status, if patients were not directly available. All included patients or their relatives gave their oral approval for data analysis and participation in the survey. The following data was collected with the survey: Quality of life (QoL) was graded with the Short Form-8 physical and mental component scores (SF-8 PCS and MCS) [15]. The actual mobility was further assessed by the Parker Mobility Score (PMS) [16]. Pain was rated with the numeric rating scale (NRS) [17]. Personal data were anonymized before analysis. The study was approved by the local ethics committee (Reference: 837.140.17 (10974)).

We tested continuous data for normal distribution using the Kolmogorov Smirnov test. Descriptive statistics in normally distributed data was described as mean and standard deviation. In non-normally distributed data, median and the 25th and 75th interquartile ranges (IQR) were calculated. Different groups were compared using the non-paired student’s t test (normally distributed data) and the Mann-Whitney-U test (non-normally distributed data). Nominal groups were compared using the chi-square test. Survival analysis was computed according to Kaplan-Meier. A p value of ≤0.05 was considered to be significant. Statistical analysis was performed using SPSS software (IBM SPSS Statistics for Windows, Version 23; IBM Corp, Armonk, NY, USA).

## Results

### All patients

**Fig 2:**
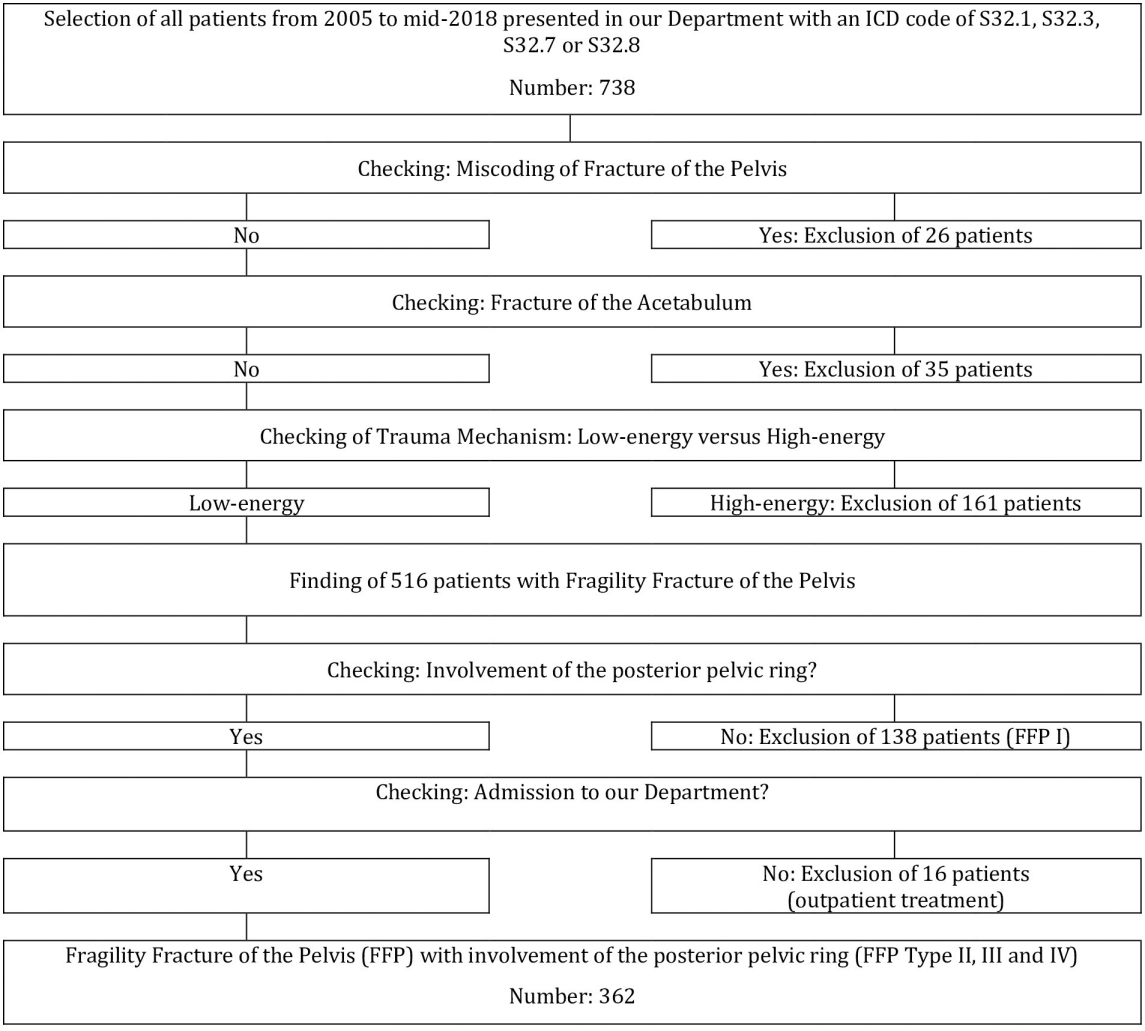
Flowchart of excluded and included study patients.

Overall, 362 patients with an FFP type II, III and IV were included (Fig 2).

There were 314 women (86.7%) and 48 men (13.3%). Tables 1 and 2 show data on demographics, type of treatment, LoS, in-hospital complications, mobility and distination at discharge, mortality and QoL at follow up, depending on FFP-classification (Table 1) and on the type of treatment (Table 2).

**Table 1:** Demographics, type of treatment, LoS, mortality and data at follow up of all patients, depending on FFP-classification.

	FFP Type II—IV	FFP Type II	FFP Type III-IV	p-value
Number of patients	362	238	124	
Age (median)	81	82	77	**<0.001**
Women (n, %)	314 (86.7)	204 (85.7)	110 (88.7)	
Men (n, %)	48 (13.3)	34 (14.3)	14 (11.3)	0.425
Patients with comorbidities (n, %)—See also Table 3	334 (92.3)	223 (93.7)	111 (89.5)	0.158
Patients with two or more comorbidities (n, %)	191 (52.8)	131 (55.0)	60 (48.4)	0.229
Conservative treatment (n, %)	224 (61.9)	186 (78.2)	38 (30.6)	
Operative treatment (n, %)	138 (38.1)	52 (21.8)	86 (69.4)	**<0.001**
Median length of hospital stay (days)	12	10	15	**<0.001**
**General in-hospital complications (n, %)**	101 (27.9)	53 (22.3)	48 (38.7)	**0.001**
Urinary tract infection	67 (18.5)	32 (13.4)	35 (28.2)	**0.001**
Pneumonia	19 (5.2)	11 (4.6)	8 (6.5)	0.459
Cardiovascular	9 (2.5)	8 (3.4)	1 (0.8)	0.127
Bedsore	17 (4.7)	10 (4.2)	7 (5.6)	0.538
Thrombosis	5 (1.4)	2 (0.8)	3 (2.4)	0.222
Lung embolism	2 (0.6)	1 (0.4)	1 (0.8)	0.638
**Mobility at discharge (n, %)**				
Ward	194 (53.6)	143 (60.1)	51 (41.1)	
Room	57 (15.7)	43 (18.1)	14 (11.3)	
Transfer	75 (20.7)	41 (17.2)	34 (27.4)	
Bedridden	17 (4.7)	4 (1.7)	13 (10.5)	
Not documented	15 (4.1)	5 (2.1)	10 (8.1)	
Death in hospital	4 (1.1)	2 (0.8)	2 (1.6)	**<0.001**
**Mobility at discharge (n, %)**				
Walkers	251 (73.2)	186 (80.5)	65 (58.0)	
Non walkers	92 (26.8)	45 (19.5)	47 (42.0)	**0.001**
**Destination at discharge (n, %)**				
Home	143 (39.5)	107 (45.0)	36 (29.0)	
Geriatrics	112 (30.9)	67 (28.1)	45 (36.3)	
Rehabilitation	73 (20.1)	49 (20.6)	24 (19.4)	
Hospital	16 (4.4)	8 (3.4)	8 (6.5)	
Not documented	14 (3.9)	5 (2.1)	9 (7.3)	
Death in hospital	4 (1.1)	2 (0.8)	2 (1.6)	**0.034**
**Destination at discharge (n, %)**				
Independent	143 (41.6)	107 (46.4)	36 (31.9)	
Dependent	201 (58.4)	124 (53.6)	77 (68.1)	**0.011**
Mortality in hospital (%)	1.1	0.8	1.6	0.505
One-year mortality (%)	15.1	14.2	16.9	0.462
Two-year mortality (%)	23.3	21.6	26.6	
Five-year mortality (%)	55.1	59.1	47.2	
Overall mortality (%)				0.127
Mortality before follow up (n, %)	126 (34.8)	87 (36.6)	39 (31.5)	
Lost to follow up (n, %)	32 (8.8)	16 (6.7)	16 (12.9)	
Follow up (n, %)	203 (56.1)	135 (56.7)	68 (54.8)	
Follow up of surviving patients (n, %)	203 (86.0)	135 (89.4)	68 (80.0)	
Mean follow up time (weeks)	164.3	154.3	184.1	
**Patients with PCS and MCS (n, %)**	114/203 (56.2)	67/135 (49.6)	47/68 (69.1)	
Median SF-8 physical (PCS)	32.31	32.21	32.56	0.777
Min SF-8 physical	15.03	16.34	15.03	
Max SF-8 physical	62.51	62.51	56.68	
Median SF-8 mental (MCS)	54.42	54.41	54.43	0.738
Min SF-8 mental	17.71	19.16	17.71	
Max SF-8 mental	69.22	68.44	69.22	
**Patients with PMS (n, %)**	202/203 (99.5)	134/135 (99.3)	68/68 (100.0)	
Median PMS	5	5	5	0.203
Min PMS	0	0	0	
Max PMS	9	9	9	
**Patients with NRS (n, %)**	198/203 (97.5)	133/135 (98.5)	65/68 (95.6)	0.477
Median NRS	3	3	4	
Min NRS	0	0	0	
Max NRS	10	10	10	

P-values below 0.05 are shown in bold.

**Table 2:** Demographics, LoS, mortality and data at follow up of all patients, depending on type of treatment.

	FFP Type II—IV	FFP Type II-IV conservative	FFP Type II-IV operative	p-value
Number of patients	362	224 (61.9)	138 (38.1)	
Age (median)	81	82	79	**0.001**
Women (n, %)	314 (86.7)	191 (85.7)	123 (89.1)	
Men (n, %)	48 (13.3)	33 (14.7)	15 (10.9)	0.293
Patients with comorbidities (n, %)—See also Table 3	334 (92.3)	207 (92.4)	127 (92.0)	0.186
Patients with two or more comorbidities (n, %)	191 (52.8)	124 (55.4)	67 (48.6)	0.208
Median length of hospital stay (days)	12	8	18	**<0.001**
**General in-hospital complications (n, %)**	101 (27.9)	51 (22.8)	50 (36.2)	**0.006**
Urinary tract infection	67 (18.5)	31 (13.8)	36 (26.1)	**0.004**
Pneumonia	19 (5.2)	12 (5.4)	7 (5.1)	0.906
Cardiovascular	9 (2.5)	5 (2.2)	4 (2.9)	0.470
Bedsore	17 (4.7)	6 (2.7)	11 (8.0)	**0.021**
Thrombosis	5 (1.4)	1 (0.4)	4 (2.9)	0.052
Lung embolism	2 (0.6)	1 (0.4)	1 (0.7)	0.729
**Destination at discharge (n, %)**				
Home	143 (39.5)	105 (46.9)	38 (27.5)	
Geriatrics	112 (30.9)	63 (28.1)	49 (35.5)	
Rehabilitation	73 (20.1)	35 (15.6)	38 (27.5)	
Hospital	16 (4.4)	8 (3.6)	8 (5.8)	
Not documented	14 (3.9)	9 (4.0)	5 (3.6)	
Death in hospital	4 (1.1)	4 (1.8)	0 (0.0)	**0.001**
**Destination at discharge (%)**				
Independent	143 (41.6)	105 (49.8)	38 (28.6)	
Dependent	201 (58.4)	106 (50.2)	95 (71.4)	**<0.001**
**Mobility at discharge (n, %)**				
Ward	194 (53.6)	141 (62.9)	53 (38.4)	
Room	57 (15.7)	36 (16.1)	21 (15.2)	
Transfer	75 (20.7)	24 (10.7)	51 (37.0)	
Bedridden	17 (4.7)	10 (4.5)	7 (5.1)	**<0.001**
Not documented	15 (4.1)	9 (4.0)	6 (4.3)	
Death in hospital	4 (1.1)	4 (1.8)	0 (0.0)	
**Mobility at discharge (n, %)**				
Walking	251 (73.2)	177 (83.9)	74 (56.1)	
Non walking	92 (26.8)	34 (16.1)	58 (43.9)	**<0.001**
Mortality in hospital (%)	1.1	1.8	0.0	0.114
One-year mortality (%)	15.1	18.3	9,9	**0.032**
Two-year mortality (%)	23.3	27,7	16	
Five-year mortality (%)	55.1	64,3	41	
Overall mortality (%)				**<0.001**
Mortality before follow up (n, %)	126 (34.8)	93 (41.5)	33 (23.9)	
Lost to follow up (n, %)	32 (8.8)	17 (7.6)	15 (10.9)	
Follow up (n, %)	32 (8.8)	114 (50.9)	89 (64.5)	
Follow up of surviving patients (n %)	203 (56.1)	114 (87.0)	89 (84.8)	
Mean follow up time (weeks)	164.3	150.3	182.1	
**Patients with PCS and MCS (n, %)**	114/203 (56.2)	56/114 (49.1)	58/89 (65.2)	
Median SF-8 physical (PCS)	32.31	32.86	31.98	0.638
Min SF-8 physical	15.03	15.03	17.34	
Max SF-8 physical	62.51	62.51	56.68	
Median SF-8 mental (MCS)	54.42	53.99	54.42	0.672
Min SF-8 mental	17.71	19.16	17.71	
Max SF-8 mental	69.22	68.44	69.22	
**Patients with PMS (n, %)**	202/203 (99.5)	113/114 (99.1)	89/89 (100.0)	
Median PMS	5	5	5	0.285
Min PMS	0	0	0	
Max PMS	9	9	9	
**Patients with NRS (n, %)**	198/203 (97.5)	200/203 (98.5)	87/89 (97.8)	
Median NRS	3	1	4	**0.013**
Min NRS	0	0	0	
Max NRS	10	10	10	

P-values below 0.05 are shown in bold.

238 patients had an FFP type II (65.7%) and 124 a type III or IV (34.3%). Median age of all patients was 81 years. Median age of patients with FFP type III and IV was significantly lower than of patients with type II (p<0.001). 334 patients (92.3%) presented with comorbidities, 191 patients (52.8%) with two or more comorbidities. Type and number of comorbidities, depending on FFP-classification and on type of treatment, are depicted in Table 3. Patients with FFP type III and IV suffered less often from cardiovascular diseases (p = 0.042), from diabetes mellitus (p = 0.05) and from dementia (p = 0.049). Operated patients, had less often dementia (p = 0.003) but more often osteoporosis (p<0.001).

**Table 3:** Type and number of comorbidities in all patients, depending on FFP-classification and on type of treatment.

**FFP Type**	**Type II-IV**	**Type II**	**Type III and IV**	**p-value**
Number of patients	362	238	124	
Patients with comorbidities (n, %)	334 (92.3)	223 (93.7)	111 (89.5)	0.158
Patients with two or more comorbidities (n, %)	191 (52.8)	131 (55.0)	60 (48.4)	0.229
cardiovascular disease	303	206	97	0.042
malignancy	80	48	32	0.22
diabetes mellitus	83	62	21	**0.05**
dementia	58	45	13	**0.049**
pulmonary disease	37	26	11	0.541
rheumatoid arthritis	23	14	9	0.611
osteoporosis	191	117	74	0.057
**Type of treatment**	**All Patients**	**non-operative**	**operative**	**p-value**
Number of patients	362	224	138	
Patients with comorbidities (n, %)	334 (92.3)	207 (92.4)	127 (92.0)	0.895
Patients with two or more comorbidities (n, %)	191 (52.8)	124 (55.4)	67 (48.6)	0.208
cardiovascular disease	303	185	118	0.465
malignancy	80	49	31	0.896
diabetes mellitus	83	56	27	0.232
dementia	58	46	12	**0.003**
pulmonary disease	37	22	15	0.749
rheumatoid arthritis	23	14	9	0.918
osteopororsis	191	103	88	**<0.001**

P-values below 0.05 are shown in bold.

224 patients (61.9%) were treated non-operatively and 138 patients (38.1%) operatively. Type and localization of the operative stabilization, depending on the FFP-classification, are represented in Table 4.

**Table 4:** Type and number of operative procedures on posterior and anterior pelvis in 138 patients.

FFP Type	FFP II-IV	FFP II	FFP III-IV
Number of patients	138	52	86
**Posterior Pelvis: number of procedures**	**136**	**50**	**86**
Transsacral bar with bilateral IS screws	36	6	30
Transsacral bar	29	9	20
IS Screws unilateral	30	25	5
Transsacral bar with unilateral IS screw	16	7	9
Plate and screw osteosynthesis ilium	10	0	10
IS screws bilateral	7	2	5
IS screw with plate and screw osteosynthesis ilium	2	0	2
Transsacral bar with plate and screw osteosynthesis ilium	2	0	2
Transiliac bridging plate osteosynthesis with bilateral IS screws	1	0	1
Internal fixator	1	1	0
Internal fixator with bilateral IS screws	1	0	1
Lumbopelvic fixation with bilateral IS screws	1	0	1
**Anterior pelvis: number of procedures**	**88**	**39**	**49**
Unilateral retrograde transpubic screw	49	29	19
Plate and screw osteosynthesis	27	5	22
Bilateral transpubic screw	8	4	4
Retrograde transpubic screw and plate and screw osteosynthesis	2	1	2
Plate and external fixator	2	0	2
**Localisation of operative stabilization**			
Posterior and anterior pelvic stabilization	86	37	49
Posterior pelvis only without anterior pelvis	50	13	37
Anterior pelvis only without posterior pelvis	2	2	0

IS = iliosacral.

**Fig 3:**
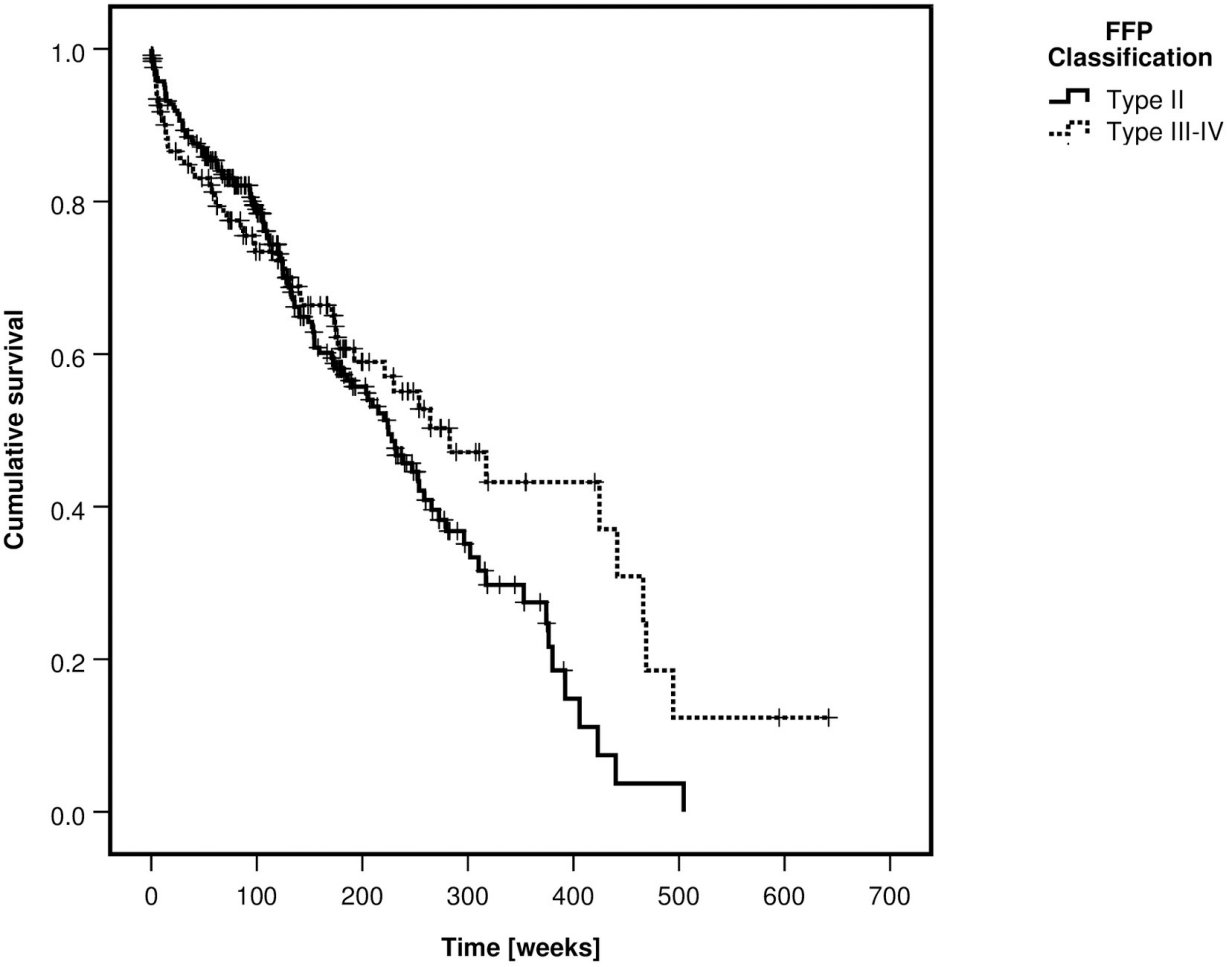
Kaplan-Meier curve of cumulative survival, depending on FFP-classification—All patients.

**Fig 4:**
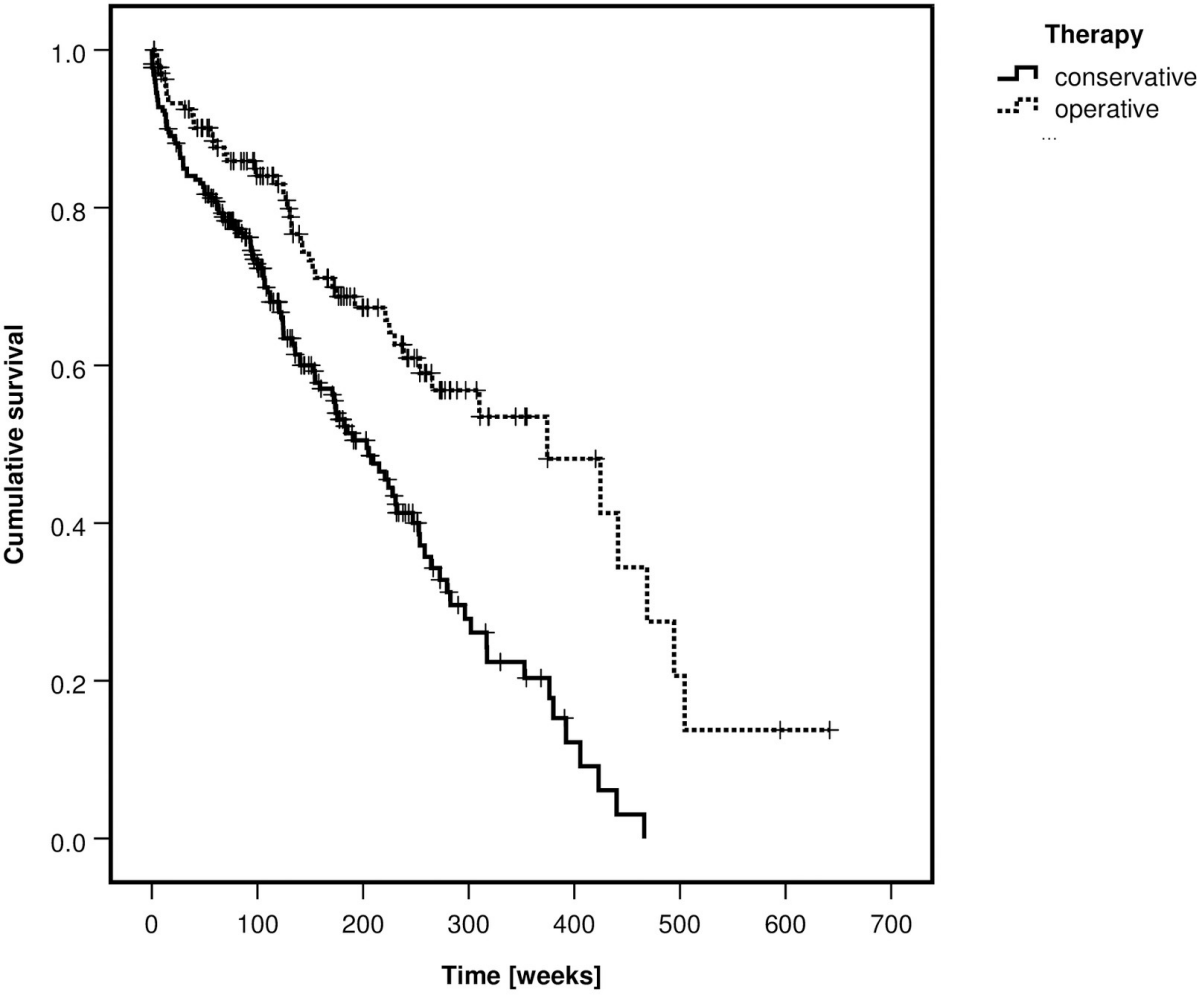
Kaplan-Meier curve of cumulative survival, depending on type of treatment—All patients.

Patients with FFP type III and IV were more often treated operatively than patients with type II (p<0–001). One-year mortality was 15.1%, two-year mortality 23.3% and five-year mortality 55.1%. Overall mortality was not different between fracture classes (p = 0.127) but significantly lower in the operative group (p<0.001). Kaplan-Meier curves of the survival rates, depending on the FFP-classification and on the type of treatment are depicted in Figs 3 and 4.

The median LoS was significantly longer in patients with FFP type III and IV (p<0.001) and in the operatively treated group (p<0.001). In the operative group, the median postoperative LoS of FFP type II was 12 days and of FFP type III and IV 13 days. There were more general in-hospital complications in patients with FFP type III and IV (p = 0.001) and in the operatively treated group (p = 0.006). The vast majority of patients (78.5%) lived in a domestic environment before hospital admission. Only half of them could return home at discharge. Pre-hospital environment and destination at discharge of all patients are shown in Table 5.

**Table 5:** Pre-hospital environment and distination and discharge of all patients, depending on type of treatment.

**FFP Type II—IV**	**Pre-hospital**	**Discharge**	**p-value**
All patients (n, %)	362 (100)	362 (100)	
Home	284 (78.5)	143 (39.5)	
Geriatrics	38 (10.5)	112 (30.9)	
Rehabilitation	2 (0.6)	73 (20.1)	
Hospital	12 (3.3)	16 (4.4)	**<0.001**
Not documented	26 (7.9)	14 (3.9)	
Died in hospital	0 (0.0)	4 (1.1)	
Independent	284 (85.1)	143 (41.6)	
Dependent	52 (14.9)	201 (58.4)	**<0.001**
**Type of treatment**	**Pre-hospital**	**Discharge**	**p-value**
All patients (n, %)	224 (100)	224 (100)	
Home	173 (77.2)	105 (46.9)	
Geriatrics	26 (11.6)	63 (28.1)	
Rehabilitation	0 (0.0)	35 (15.6)	
Hospital	7 (3.1)	8 (3.6)	**< 0.001**
Not documented	7 (3.1)	9 (4.0)	
Died in hospital	0 (0.0)	4 (1.8)	
Independent	173 (84.0)	105 (49.3)	
Dependent	33 (16.0)	106 (50.7)	**<0.001**
**FFP Type II—IV operative**	**Pre-hospital**	**Discharge**	**p-value**
All patients	138 (100)	138 (100)	
Home (%)	111 (80.4)	38 (27.5)	
Geriatrics (%)	12 (8.7)	49 (35.5)	
Rehabilitation (%)	2 (1.4)	38 (27.5)	
Hospital (%)	5 (3.6)	8 (5.8)	**<0.001**
Not documented (%)	8 (5.8)	5 (3.6)	
Died in hospital (%)	0 (0.0)	0 (0.0)	
Independent (%)	111 (85.4)	38 (28.6)	
Dependent (%)	19 (14.6)	95 (71.4)	**<0.001**

P-values below 0.05 are shown in bold.

More patients with FFP type II (p = 0.011) and of the non-operativey treated group (p<0.001) returned home after discharge. Mobility at discharge was significantly better in patients with FFP type II (p<0.001) and in the non-operativenon-operativey treated group (p<0.001). Non-operativenon-operative126 patients (34.8%) had died before follow up by telephone call. 32 were lost to follow up. 203 patients (56.1%) took part in the survey. The follow up rate of surviving patients was 86.0%. Mean follow up time was 38 months. At follow up, SF-8 PCS, SF-8 MCS and PMS did not differ between the fracture classes and the treatment groups. NRS was not different between the fracture classes, but scored higher in the operative group (p = 0.013). See also Tables 1 and 2.

### FFP Type II

238 patients had FFP type II. Demographics of these patients, type of treatment, LoS, in-hospital complications, mobility and destination at discharge, mortality and QoL at follow up, depending on the type of treatment are shown in Table 6.

**Table 6:** Demographics, LoS, mortality, situation at discharge and at follow up of FFP type II patients, depending on type of treatment.

Type of treatment	All patients	non-operative	operative	p-value
Number of patients (n, %)	238 (100)	186 (78.2)	52 (21.8)	
Age (median)	82	82.5	79	**0.006**
Women (n, %)	204 (85.7)	159 (85.5)	45 (86.5)	
Men (n, %)	34 (14.3)	27 (14.5)	7 (13.5)	0.848
Patients with comorbidities (n, %)	223 (93.7)	174 (93.5)	49 (94.2)	0.787
Patients with two or more comorbidities (n, %)	131 (55.0)	104 (55.9)	27 (51.9)	0.609
Median length of hospital stay (days)	10	9	17	**<0.001**
Minimum	0	0	3	
Maximum	92	28	92	
IQR	7–14.8	6–12	14.8–25	
General in-hospital complications (n, %)	53 (22.3)	39 (19.8)	14 (27.5)	0.362
Surgery-related complications (n, %)	n.a.	n.a.	12 (23.1)	
Surgical revisions (n, %)			9 (17.3)	
**Mobility at discharge (n, %)**				
Ward	143 (60.1)	121 (65.1)	22 (42.3)	
Room	43 (18.1)	35 (18.8)	8 (15.4)	
Transfer	41 (17.2)	23 (12.4)	18 (34.6)	
Bedridden	4 (1.7)	3 (1.6)	1 (1.9)	
Not documented	5 (2.1)	2 (1.1)	3 (5.8)	
Death in hospital	2 (0.8)	2 (1.1)	0 (0.0)	
**Mobility at discharge (n, %)**				
Walker	186 (80.5)	156 (85.7)	30 (61,2)	
Non-walker	45 (19.5)	26 (14.3)	19 (38.8)	**<0.001**
**Destination at discharge (n, %)**				
Home	107 (45.0)	88 (47.3)	19 (36.5)	
Geriatrics	67 (28.1)	56 (30.1)	11 (21.2)	
Rehabilitation	49 (20.6)	31 (16.7)	18 (34.6)	
Hospital	8 (3.4)	5 (2.7)	3 (5.8)	**0.022**
Not documented	5 (2.1)	4 (2.2)	1 (1.9)	
Death in hospital	2 (0.8)	2 (1.1)	0 (0.0)	
**Destination at discharge (n, %)**				
Independent	167 (57.4)	88 (48.8)	19 (37.3)	
Dependent	124 (42.6)	92 (51.2)	32 (62.7)	0.141
**Mobility at discharge (n, %)**				
Walking	186 (80.5)	156 (85.7)	30 (61.2)	
Non walking	45 (19.5)	26 (14.3)	19 (38.8)	**<0.001**
Mortality in hospital (%)	1.1	1.1	0.0	0.453
One-year mortality (%)	14.2	15.9	8.0	0.154
Two-year mortality (%)	21.6	25.3	8.0	
Five-year mortality (%)	59.1	67.1	33.2	
Overall mortality				**0.001**
Mortality before follow up (n, %)	87 (36.6)	76 (40.9)	11 (21.2)	
Lost to follow up (n, %)	16 (6.7)	10 (5.4)	6 (11.5)	
Follow up (n, %)	135 (56.7)	100 (53.8)	35 (67.3)	
Follow up of surviving patients (n, %)	135 (89.4)	100 (90.9)	35 (85.4)	
Median follow up time (weeks)	164	117	119.7	
**Patients with PCS and MCS (n, %)**	67/135 (49.6)	51/100 (51.0)	16/35 (45.7)	
Median SF-8 physical (PCS)	32.21	33.41	29.39	0.734
Min SF-8 physical	16.34	16.34	17.34	
Max SF-8 physical	62.51	62.51	56.68	
Median SF-8 mental (MCS)	54.41	53.55	55.42	0.938
Min SF-8 mental	19.16	19.16	19.83	
Max SF-8 mental	68.44	68.44	65.17	
**Patients with PMS (n, %)**	134/135 (99.3)	99/100 (99.0)	35/35 (100.0)	
Median PMS	5	5	6	0.285
Min PMS	0	0	0	
Max PMS	9	9	9	
**Patients with NRS (n, %)**	133/135 (98.5)	98/100 (98.0)	35/35 (100.0)	
Median NRS	3	1	4	**0.024**
Min NRS	0	0	0	
Max NRS	10	10	9	

P-values below 0.05 are shown in bold.

**Fig 5:**
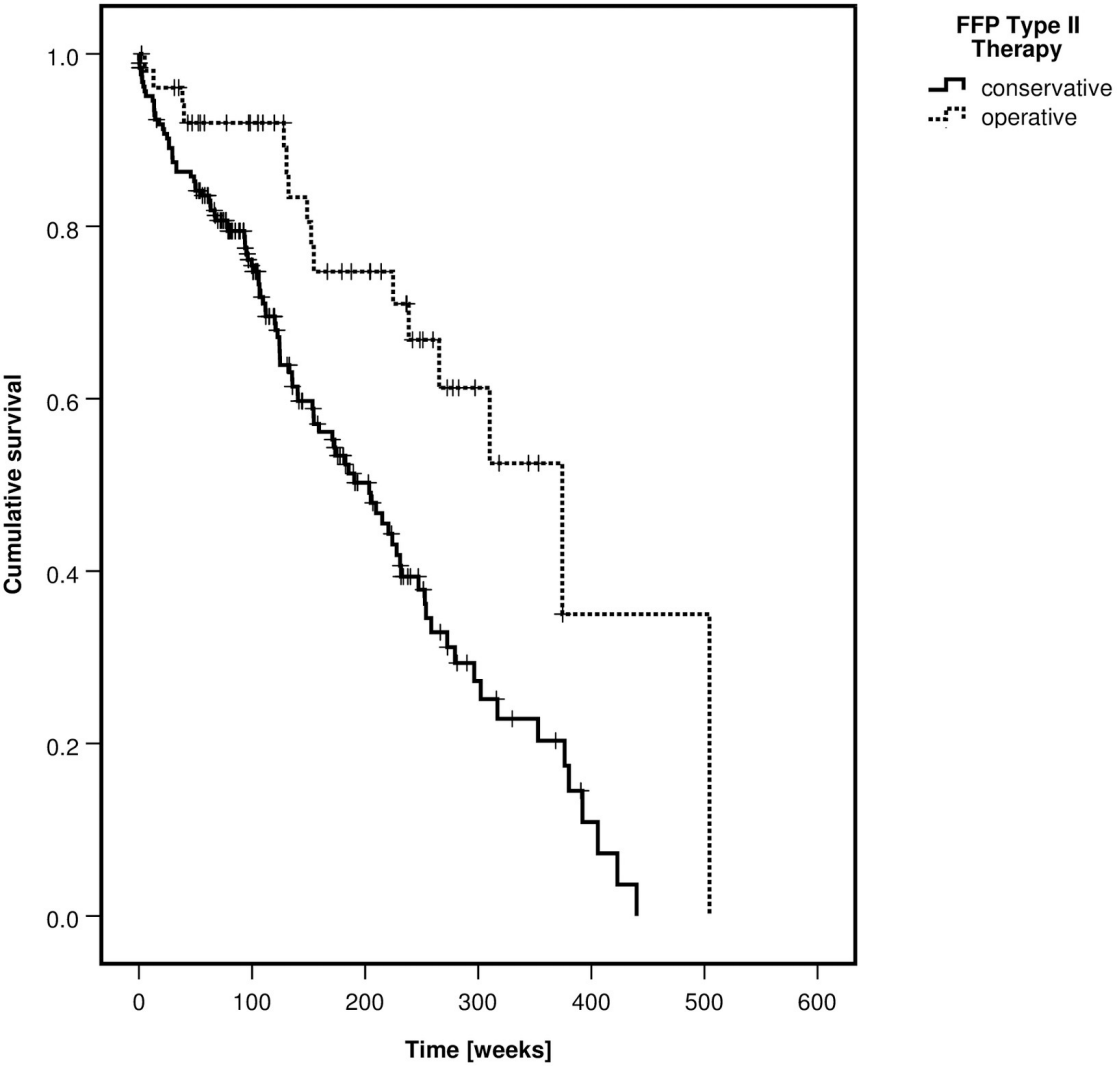
Kaplan-Meier curve of cumulative survival, depending on type of treatment—FFP type II patients.

There were 204 women (85.7%) and 34 men (14.3%). Their median age was 82 years. The median age of the operative group was 3.5 years lower than of the non-operative group (p = 0.006). 186 patients were treated non-operatively (78.2%) and 52 operatively (21.8%). The type of operative treatment is presented in Table 4. Iliosacral (IS) screw osteosynthesis was the most frequent stabilization technique, followed by transsacral bar osteosynthesis. Overall mortality was significantly lower in the operative group (p = 0.001). A Kaplan-Meier curve depicts the survival rates of the non-operative and operative group (Fig 5).

The median LoS was significantly longer in the operatively treated group (p<0.001). The number of general in-hospital complications was not different between the groups (p = 0.362). The non-operative group was significantly more mobile than the operative group at discharge (p<0.001), but independency did not differ (p = 0.141).

135 of 151 surviving patients (89.4%) took part in the survey after a mean follow-up time of 36 months. SF-8 PCS and SF-8 MCS was available for 67 patients (49.6%). There was no significant difference between the values of the non-operative and operative group (p = 0.734 for SF-8 PCS and p = 0.938 for SF-8 MCS). PMS was calculated for 134 patients (99.3%). There was no difference between the PMS of both groups (p = 0.285). NRS was available in 133 patients (98.5%). NRS was significantly higher in the operative group (p = 0.024).

### FFP Type III and IV

There were 124 FFP type III and IV. Demographics of these patients, type of treatment, LoS, in-hospital complications, mobility and destination at discharge, mortality and QoL at follow up, depending on the type of treatment are shown in Table 7.

**Table 7:** Demographics, LoS, mortality, situation at discharge and at follow up of FFP type II patients, depending on type of treatment.

Type of treatment	All patients	non-operative	operative	p-value
Number of patients (%)	124 (100)	38 (30.6)	86 (69.4)	
Age (median)	77	74.5	77.5	0.427
Women (n, %)	110 (88.7)	32 (84.2)	78 (90.7)	
Men (n, %)	14 (11.3)	6 (15.8)	8 (9.3)	0.293
Patients with comorbidities (n, %)	111 (89.5)	33 (86.8)	78 (90.7)	0.705
Patients with two or more comorbidities (n, %)	60 (48.4)	20 (52.6)	40 (46.5)	0.53
Median length of hospital stay (days)	15	7.5	18	**<0.001**
Minimum	1	1	6	
Maximum	68	29	68	
IQR	10–21	5–11.8	14–25	
Patients with general complications (n, %)	48 (38.7)	12 (31.6)	36 (41.9)	0.279
Patients with surgery-related complications (n, %)	n.a.	n.a.	24 (27.9)	
Surgical revisions (n, %)			18 (20.9)	
**Mobility at discharge (n, %)**				
Ward	51 (41.1)	20 (52.6)	31 (36.0)	
Room	14 (11.3)	1 (2.6)	13 (15.1)	
Transfer	34 (27.4)	1 (2.6)	33 (38.4)	
Bedridden	13 (10.5)	7 (18.4)	6 (7.0)	
Not documented	10 (8.1)	7 (18.4)	3 (3.5)	
Death in hospital	2 (1.6)	2 (5.3)	0 (0.0)	
**Mobility at discharge (n, %)**				
Walking	65 (58.0)	21 (72.4)	44 (53.0)	
Non walking	47 (42.0)	8 (27.6)	39 (47.0)	0.068
**Destination at discharge (n, %)**				
Home	36 (29.0)	17 (44.7)	19 (22.1)	
Geriatrics	45 (36.3)	7 (18.4)	38 (44.2)	
Rehabilitation	24 (19.4)	4 (10.5)	20 (23.3)	
Hospital	8 (6.5)	3 (7.9)	5 (5.8)	**0.007**
Not documented	9 (7.3)	5 (13.2)	4 (4.7)	
Death in hospital	2 (1.6)	2 (5.3)	0 (0.0)	
**Destination at discharge (n, %)**				
Independent	36 (29.2)	17 (54.8)	19 (23.2)	
Dependent	77 (70.8)	14 (45.2)	63 (76.8)	**0.001**
Mortality in hospital (%)	1.1	5.3	0.0	**0.032**
One-year mortality (%)	15.3	26.3	10.5	**0.005**
Two-year mortality (%)	23.4	34.2	18.6	
Five-year mortality (%)	34.7	39.5	32.6	
Overall mortality				**0.023**
Mortality before follow up (n, %)	39 (31.5)	17 (44.7)	22 (25.6)	
Lost to follow up (n, %)	17 (13.7)	7 (18.4)	9 (10.5)	
Follow up (n, %)	68 (54.8)	14 (36.8)	54 (62.8)	
Follow up of surviving patients (n, %)	68 (80.0)	14 (66.6)	54 (85.7)	
Median follow up time (weeks)	170	167	132.6	
**Patients with PCS and PMS (n, %)**	47/68 (69.1)	5/14 (35.7)	42/54 (77.8)	
Median SF-8 physical (PCS)	32.56	23.38	32.64	0.429
Min SF-8 physical	15.03	15.03	18.59	
Max SF-8 physical	56.68	56.68	55.5	
Median SF-8 mental (MCS)	54.43	57.48	54.27	0.446
Min SF-8 mental	17.71	43.95	17.71	
Max SF-8 mental	69.22	66.17	69.22	
**Patients with PMS (n, %)**	68/68 (100.0)	14/14 (100.0)	54/54 (100.0)	
Median PMS	5	4.5	5	0.63
Min PMS	0	0	0	
Max PMS	9	9	9	
**Patients with NRS (n, %)**	65/68 (95.6)	13/14 (92.9)	52/54 (96.3)	
Median NRS	4	2	4	0.278
Min NRS	0	0	0	
Max NRS	10	6	10	

P-values below 0.05 are shown in bold.

**Fig 6:**
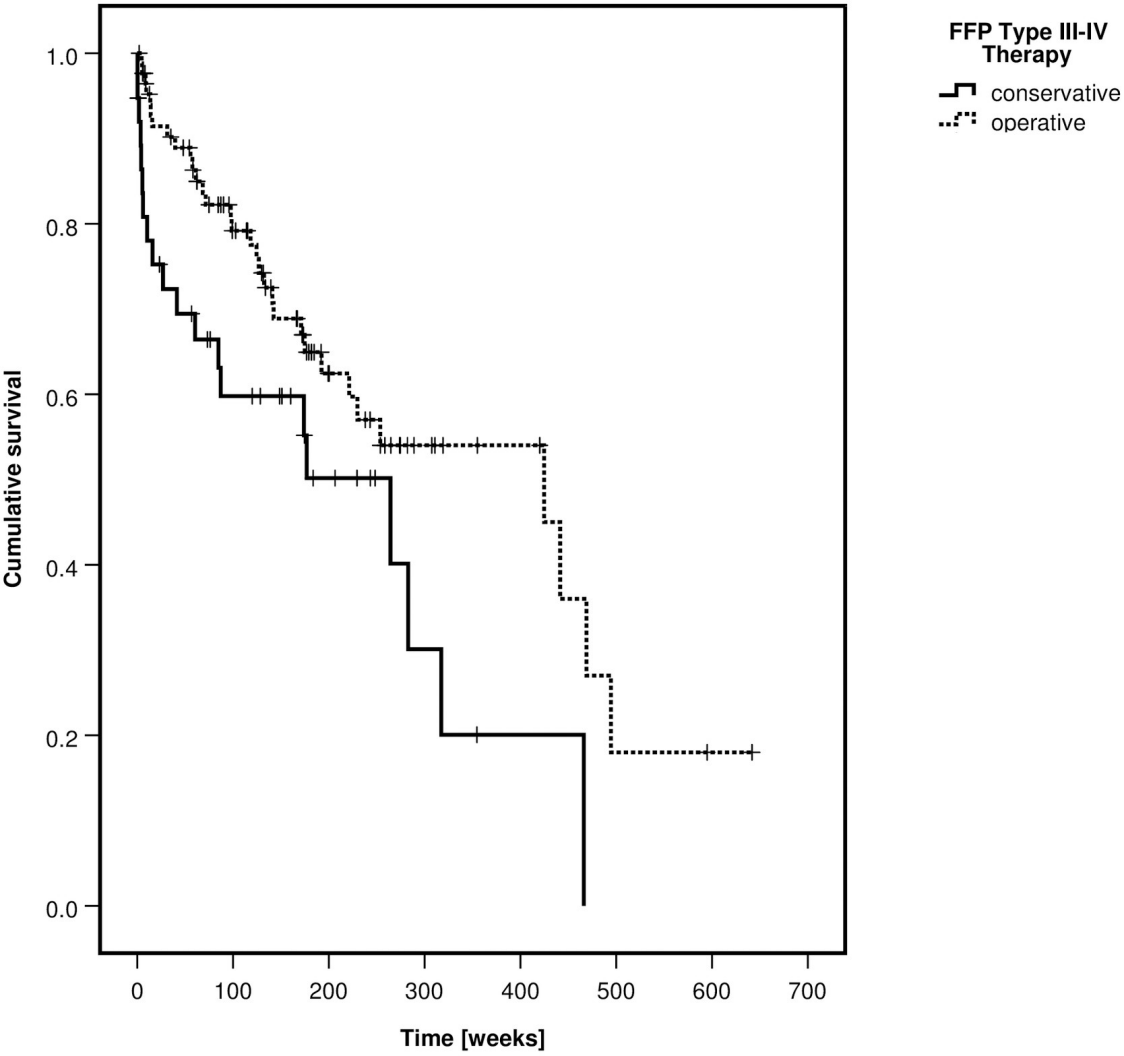
Kaplan-Meier curve of cumulative survival, depending on type of treatment—FFP type III and type IV patients.

There were 110 women (88.7%) and 14 men (11.3%). 30 patients had an FFP type III and 94 a type IV. The median age of all patients was 77 years without difference between the non-operative and operative group (p = 0.427). 38 patients were treated nonoperatively (30.6%) and 86 operatively (69.4%). The type of operative treatment is presented in Table 4. Transsacral bar osteosynthesis was the most frequent stabilization technique, followed by plate and screw osteosynthesis of the ilium. Overall mortality was significantly lower in the operative group (p = 0.023). A Kaplan-Meier curve depicts the survival rates of the non-operative and operative group (Fig 6).

The median LoS was significantly longer in the operatively treated group (p<0.001). The number of general in-hospital complications did not differ between the groups (p = 0.279). The mobility at discharge was not significantly different between the groups (p = 0.068). Nevertheless, more patients were independent at discharge in the non-operative group (p = 0.001).

68 of 85 surviving patients (80.0%) took part in the survey after a mean follow-up time 43 months. SF-8 PCS and SF-8 MCS were available for 47 patients (69.1%). The median SF-8 PCS (p = 0.429) and SF-8 MCS (p = 0.446) did not show a significant difference between the groups. PMS was available in all 68 patients (100%). There was no difference between the groups (p = 0.63). NRS was given in 65 patients (95.6%) without difference between the groups (p = 0.278). Further data are presented in Table 7.

## Discussion

Fragility fractures of the pelvis (FFP) are an increasing clinical entity, for which no treatment guidelines exist. In this retrospective observational study, we primarily aimed at investigating differences in mortality depending on fracture classification and type of treatment. We further investigated LoS, in-hospital complications, mobility and destination at discharge and QoL at follow up as secondary endpoints. The most important finding was that operatively treated patients had lower mortality despite longer LoS and more in-hospital complications than patients, who were treated non-operatively. Patients with a non-operative treatment had a better mobility and were more independent at discharge, but their SF-8 PCS and MCS, PMS and NRS did not differ from the operative group at follow up.

Recommendations for operative treatment were those descibed in our original publication of 2013 [7]. Age, dementia, type and number of comorbidities and osteoporosis did not influence our advice for surgical treatment. Nevertheless, not every patient followed our recommendations. The subjective estimation of their actual status and the opinion of the relatives may have influenced the decision of many patients. Anxiety for surgery, faith in the benefits of non-operative treatment and frailty may have driven them towards a non-operative treatment, despite an advise for surgical treatment. The time needed for a decision explains why surgery only was performed at a median of 6 days after admission. 21.8% of patients with FFP type II and 69.4% of patients with FFP type III and IV were treated operatively.

Patients with operative treatment left the hospital at a median of 12 days after surgery. 62.3% received a stabilization of the posterior and anterior pelvis. 12 days after surgery, patients still had postoperative pain and their surgical wounds were not completely healed. This explains why mobilization at discharge was impaired and patients more frequently needed support from third parties than non-operative patients. The operative procedure led to a decline of the general condition in the perioperative phase. At follow up, operatively treated patients showed good recovery, which resulted in a lower mortality than in non-operative patients.

The included patients had a median age of 81, the vast majority of them being female. This data is consistent with other recent publications [14, 18–21]. Patients with FFP type III and IV were younger than patients with FFP type II. The younger, the more mobile the patients are. Consequently, higher stresses exist on the posterior pelvis, which ultimately leads to more unstable fracture types [22]. Instability is regarded as an indication for surgical stabilization, but there is no proof of superior outcome until now [23, 24].

In patients of old age, the decision to operate should be taken after careful consideration. Over 90% of our patients presented with at least one, half of them with two or more comorbidities. This data is consistent with numbers in literature. In a study of Nikkel et al., 95% of 32.440 patients of 55 years of age and older, who suffered hip fractures, had at least one comborbidity [25]. In our study, the operative group was 3 years younger, had less often dementia and more often osteoporosis than the non-operative group. This may suggest that there was a bias in the decision-making on whom to operate or not, which may have influenced outcome. As mentioned above, age did not play a role in our decision-making. Dementia and osteoporosis did not influence our treatment strategy, but may have influenced outcome: whereas osteoporosis makes adequate stabilization more difficult to achieve, dementia hinders active rehabilitation.

The rate of general in-hospital complications in our study population was 27.9%, most important urinary tract infection and bedsore of any stage. These complications prove that patients before and after the operation are bedridden. van Dijk et al. registered 20.2% in-hospital complications [18]. Banierink et al. calculated a rate of 23% within 30 days after injury [26]. Median LoS and in-hospital complications were significantly higher in patients with FFP type III and IV and in the operative group. non-operativeIn our study group, patients were operated at a median of 6 days after admission. Preoperative time prolonged total LoS. A quicker decision on the type of treatment is needed for reducing LoS. In-hospital mortality was very low. There were no in-hospital deaths in the operative group. This shows that patients were well prepared for surgery and perioperative care adequate.

In patients with FFP type II, we observed some remarkable differences between the non-operative and operative group. Overall mortality was significantly lower in the operative group. Nevertheless, the observed mortality was much higher than in a reference population of the same age and living in the same region [27]. Surgery-related complications were observed in 23.1% of the operative group. This group was significantly less mobile and less independent at the time of discharge, but SF-8 PCS, SF-8 MCS and PMS were comparable at follow up. This data suggests that operatively treated patients suffer reduction of general condition in the postoperative period, but profit from better recovery, which eventually results in lower mortality.

In patients with FFP type III and IV, the differences between the non-operative and operative group were more pronounced. Overall mortality was significantly lower in the operative group. The operated patients suffered surgery-related complications in 27.9% and were less mobile and less independent at discharge. At follow up, SF-8 PCS, SF-8 MCS, PMS and NRS did not differ between the groups. Operatively treated patients with FFP type III and IV suffer from a decline in their general condition post surgery, but profit from better recovery than patients, who were selected for non-operative treatment. This eventually leads to a lower mortality in the operative group.

FFP are serious adverse event for elderly persons. Mortaility rate is 3 times as high as in a comparable population without FFP [10, 27]. There is need for a multi-disiplinary treatment of the underlying factors, which led to the FFP, in combination with treatment of the fractures [28]. Schmitz et al. found higher complication rates in operatively treated patients. Their patient-related outcome was reduced and the two-year survival of conservativaly and operatively treated patients similar [14]. Noser et al. presented high in-hospital complications and high mortality rates after surgical treatment [20]. Data are only partially comparable with our data as Schmitz et al. did not differentiate between the FFP classes and Noser et al. only looked at operated patients.

This is the first large retrospective study, which shows that operative treatment of FFP type II, III and IV provides low mortality. Notwithstanding the above, the study has several limitations. The retrospective nature involves lack of information in a minority of patients. We only could collect data from three time points: admission, discharge and survey. There were no control visits between discharge and survey. Follow up times were different for all patients and not all of them were able to participate in the survey. The lower age and the lower rate of dementia in the operated group may have influenced mortality rate. Several surgical techniques of stabilization were used and a comparison between the methods was not performed. Long-term follow up studies of elderly persons are difficult because of their enhanced mortality. Prospective studies will shed better light on the characterictics of FFP and their optimal treatment. A multicenter prospective observational study under our guidance has been set up with this goal.

## Conclusion

In this retrospective study, operative treatment of FFP type II, III and IV was connected with low mortality. Overall mortality was independent of the FFP classification. Patients with FFP were elderly persons and had several comorbidities. The decision to treat them operatively should be taken after careful consideration. Surgical treatment induces a longer LoS and higher in-hospital complication. In the early postoperative phase, operatively treated patients are less mobile and less independent than the non-operative group. At follow up, SF-8 PCS, SF-8 MCS, PMS and NRS did not differ between the groups. The data shows that operative treatment of FFP with involvement of the posterior pelvis is associated with serious early postoperative hazards. Nevertheless, mortality is low and patients obtain moderate functional results at follow up.
