# Correction: Operative treatment of fragility fractures of the pelvis is connected with lower mortality. A single institution experience

**DOI:** 10.1371/journal.pone.0258076

**Published:** 2021-09-27

**Authors:** Pol Maria Rommens, Mehdi Boudissa, Sven Krämer, Miha Kisilak, Alexander Hofmann, Daniel Wagner

There is an error in the caption of Table 7 Please see the complete, correct Table 7 caption here.

**Table 7:** Demographics, LoS, mortality, situation at discharge and at follow up of FFP type III and FFP type IV patients, depending on type of treatment.

Type of treatment	All patients	non-operative	operative	p-value
Number of patients (n, %)	238 (100)	186 (78.2)	52 (21.8)	
Age (median)	82	82.5	79	**0.006**
Women (n, %)	204 (85.7)	159 (85.5)	45 (86.5)	
Men (n, %)	34 (14.3)	27 (14.5)	7 (13.5)	0.848
Patients with comorbidities (n, %)	223 (93.7)	174 (93.5)	49 (94.2)	0.787
Patients with two or more comorbidities (n, %)	131 (55.0)	104 (55.9)	27 (51.9)	0.609
Median length of hospital stay (days)	10	9	17	**<0.001**
Minimum	0	0	3	
Maximum	92	28	92	
IQR	7–14.8	6–12	14.8–25	
General in-hospital complications (n, %)	53 (22.3)	39 (19.8)	14 (27.5)	0.362
Surgery-related complications (n, %)	n.a.	n.a.	12 (23.1)	
Surgical revisions (n, %)			9 (17.3)	
**Mobility at discharge (n, %)**				
Ward	143 (60.1)	121 (65.1)	22 (42.3)	
Room	43 (18.1)	35 (18.8)	8 (15.4)	
Transfer	41 (17.2)	23 (12.4)	18 (34.6)	
Bedridden	4 (1.7)	3 (1.6)	1 (1.9)	
Not documented	5 (2.1)	2 (1.1)	3 (5.8)	
Death in hospital	2 (0.8)	2 (1.1)	0 (0.0)	
**Mobility at discharge (n, %)**				
Walker	186 (80.5)	156 (85.7)	30 (61,2)	
Non-walker	45 (19.5)	26 (14.3)	19 (38.8)	**<0.001**
**Destination at discharge (n, %)**				
Home	107 (45.0)	88 (47.3)	19 (36.5)	
Geriatrics	67 (28.1)	56 (30.1)	11 (21.2)	
Rehabilitation	49 (20.6)	31 (16.7)	18 (34.6)	
Hospital	8 (3.4)	5 (2.7)	3 (5.8)	**0.022**
Not documented	5 (2.1)	4 (2.2)	1 (1.9)	
Death in hospital	2 (0.8)	2 (1.1)	0 (0.0)	
**Destination at discharge (n, %)**				
Independent	167 (57.4)	88 (48.8)	19 (37.3)	
Dependent	124 (42.6)	92 (51.2)	32 (62.7)	0.141
**Mobility at discharge (n, %)**				
Walking	186 (80.5)	156 (85.7)	30 (61.2)	
Non walking	45 (19.5)	26 (14.3)	19 (38.8)	**<0.001**
Mortality in hospital (%)	1.1	1.1	0.0	0.453
One-year mortality (%)	14.2	15.9	8.0	0.154
Two-year mortality (%)	21.6	25.3	8.0	
Five-year mortality (%)	59.1	67.1	33.2	** **
Overall mortality				**0.001**
Mortality before follow up (n, %)	87 (36.6)	76 (40.9)	11 (21.2)	
Lost to follow up (n, %)	16 (6.7)	10 (5.4)	6 (11.5)	
Follow up (n, %)	135 (56.7)	100 (53.8)	35 (67.3)	
Follow up of surviving patients (n, %)	135 (89.4)	100 (90.9)	35 (85.4)	
Median follow up time (weeks)	164	117	119.7	
**Patients with PCS and MCS (n, %)**	67/135 (49.6)	51/100 (51.0)	16/35 (45.7)	** **
Median SF-8 physical (PCS)	32.21	33.41	29.39	0.734
Min SF-8 physical	16.34	16.34	17.34	
Max SF-8 physical	62.51	62.51	56.68	
Median SF-8 mental (MCS)	54.41	53.55	55.42	0.938
Min SF-8 mental	19.16	19.16	19.83	
Max SF-8 mental	68.44	68.44	65.17	
**Patients with PMS (n, %)**	134/135 (99.3)	99/100 (99.0)	35/35 (100.0)	
Median PMS	5	5	6	0.285
Min PMS	0	0	0	
Max PMS	9	9	9	
**Patients with NRS (n, %)**	133/135 (98.5)	98/100 (98.0)	35/35 (100.0)	
Median NRS	3	1	4	**0.024**
Min NRS	0	0	0	
Max NRS	10	10	9	

P-values below 0.05 are shown in bold.
